# Increased early motivational response to food in adolescent anorexia nervosa revealed by magnetoencephalography

**DOI:** 10.1017/S003329172100088X

**Published:** 2022-12

**Authors:** Hugo Romero Frausto, Kati Roesmann, Isabelle A. G. Klinkenberg, Maimu A. Rehbein, Manuel Föcker, Georg Romer, Markus Junghoefer, Ida Wessing

**Affiliations:** 1 Department of Child and Adolescent Psychiatry, University Hospital Muenster, Muenster, Germany; 2 Institute for Clinical Psychology, University of Siegen, Siegen, Germany; 3 Institute for Biomagnetism and Biosignalanalysis, University Hospital Muenster, Muenster, Germany; 4 Otto Creutzfeldt Center for Cognitive and Behavioral Neuroscience, University of Muenster, Muenster, Germany

**Keywords:** Anorexia Nervosa, MEG, EEG, adolescent, food, ERP

## Abstract

**Background:**

It remains unclear to what extent reduced nutritional intake in anorexia nervosa (AN) is a consequence of a reduced motivational response to food. Although self-reports typically suggest AN patients have a reduced appetitive response, behavioral and neurophysiological measures have revealed evidence for both increased and reduced attentional biases towards food stimuli. The mechanisms influencing food perception in AN, might be clarified using time-sensitive magnetoencephalography (MEG) to differentiate the early (more automatic processing) stages from the late (more controlled) stages.

**Methods:**

MEG was recorded in 22 partially weight-restored adolescent AN patients and 29 age- and gender-matched healthy control (HC) participants during a rapid serial visual presentation paradigm using 100 high-calorie food, 100 low-calorie food, and 100 non-food pictures. Neural sources of event-related fields were estimated using the L2-Minimum-Norm method and analyzed in early (50–300 ms) and late (350–500 ms) time intervals.

**Results:**

AN patients rated high-calorie food as less palatable and reported overall less food craving than HC participants. Nevertheless, in response to food pictures AN patients showed relative increased neural activity in the left occipito-temporal and inferior frontal regions in the early time interval. No group differences occurred in the late time interval.

**Conclusions:**

MEG results speak against an overall reduced motivational response to food in AN. Instead, relative increased early food processing in the visual cortex suggests greater motivated attention. A greater appetitive response to food might be an adaptive mechanism in a state of undernourishment. Yet, this relative increased food processing in AN was no longer present later, arguably reflecting rapid downregulation.

## Introduction

Anorexia nervosa (AN) is an eating disorder characterized by an intense fear of gaining weight, reduced nutritional intake, severe weight loss, and significantly low body weight (*Diagnostic and statistical manual of mental disorders*, 5th ed.; American Psychiatric Association, 2013). Compared to older versions, the Diagnostic and statistical manual of mental disorder (DSM)-5 removed wording that implied intent on the part of the patient, indicating that patients may not necessarily exert self-control over their eating behavior. Yet, the way nutritional intake is regulated in AN remains uncertain. For example, AN patients might show a reduced motivational response to food, and/or they might (over-)regulate this response.

When asked about their subjective experience, AN patients typically report that they perceive pictured food as less pleasant or appetizing and feel less wanting compared to healthy control (HC). This pertains particularly to high-calorie food, while low-calorie food is not always rated differently (Cowdrey, Finlayson, & Park, 2013; Jiang, Soussignan, Rigaud, & Schaal, 2010; see Lloyd & Steinglass, 2018 for a review). Furthermore, AN patients describe that their general desire to eat is diminished (Holsen et al., 2012, 2014; Sanders et al., 2015). These results argue for a reduced motivational response to food in AN. However, AN patients’ tendencies towards asceticism (Fassino, Pierò, Gramaglia, & Abbate-Daga, 2004) suggest that their self-reports or feelings of appetite might be downregulated.

In this context, it is informative to consider the results of more implicit behavioral tasks thought to be less influenced by elaborate top-down processes. Depending on the task, AN patients have shown reduced, unaltered or increased responses to food (Lloyd & Steinglass, 2018). For example, AN patients were shown to be less accurate than HC in working memory tasks when food pictures are presented subliminally (Brooks et al., 2012b) or as task-irrelevant distractors (Neimeijer, Roefs, & De Jong, 2017); thus, food captured AN patients’ attention to a greater extent. In contrast, tasks designed to capture automatic approach and avoidance tendencies via motor reactions towards or away from food stimuli revealed reduced approach tendencies in AN (Neimeijer, Roefs, Glashouwer, Jonker, & de Jong, 2019; Paslakis et al., 2016; Veenstra & De Jong, 2011). Finally, when the visual exploration of food stimuli was captured by eye-tracking, both HC and AN patients directed their initial attention towards food stimuli, but in a later time interval, AN patients showed attentional disengagement (Giel et al., 2011). Taken together, behavioral tasks show food-related attention biases in AN, but in opposite directions. One explanation might be that attention shifts from an initial, bottom-up driven attention bias towards food (reflecting motivational significance) to a later, top-down driven regulatory response.

A further approach to investigate the motivational processing of food in AN is neuroimaging. In healthy participants, a meta-analysis of functional magnetic resonance imaging (fMRI) studies (Van der Laan, De Ridder, Viergever, & Smeets, 2011) located neural activation in response to food pictures compared to non-food pictures (e.g. household objects) in the insula, the orbitofrontal cortex (OFC) and the lateral occipital cortex (OCC). This meta-analysis linked the insula with taste and craving for food and the OFC with pleasantness. Moreover, activity in the lateral OCC is thought to reflect heightened attention to motivationally relevant stimuli, leading to more extensive visual processing (in the following called *motivated attention*) (Lang & Bradley, 2010; Van der Laan et al., 2011).

In AN patients, results are conflicting. One group of fMRI studies reported increased neural activity in response to food stimuli in AN patients *v.* HC, including regions associated with motivation (e.g. striatum, insula), cognitive control (e.g. frontal cortex), and visual perception and attention (e.g. OCC) (Boehm et al., 2018; Brooks et al., 2011, 2012a; Foerde, Steinglass, Shohamy, & Walsh, 2015; Gordon et al., 2001; Joos et al., 2011; Kim, Ku, Lee, Lee, & Jung, 2012; Rothemund et al., 2011; Zhu et al., 2012). However, another group of fMRI studies reported reduced neural activity in response to food stimuli in AN patients in regions associated with motivation (e.g. amygdala, insula) and visual perception and attention (OCC, parietal cortex; Gizewski *et al*., 2010; Holsen *et al*., 2012; Santel, Baving, Krauel, Muente, & Rotte, 2006). Again, these conflicting results might be explained in terms of shifting attention, whereby increased activity reflects enhanced motivated attention towards food and possibly top-down control of frontal regions, and reduced activity reflects successful downregulation.

These shifting effects of motivated attention might be disentangled by time-sensitive methods like electroencephalography (EEG) or magnetoencephalography (MEG). With respect to the processing of motivationally relevant stimuli, effects in early time intervals (*<*300 ms) are thought to reflect rather automatic motivational responses, while effects in late time intervals (*>*300 ms) are thought to reflect both in-depth processing of motivationally relevant stimuli and cognitive control (Pourtois, Schettino, & Vuilleumier, 2013). In line with this, studies investigating the time course of food processing in healthy participants using EEG have found increased amplitudes of event-related potentials (ERPs) in response to food compared to non-food stimuli in both early (*<*300 ms, Stockburger, Weike, Hamm, and Schupp, 2008) and late time windows (*>*300 ms, Sarlo, UEbel, Leutgeb, & Schienle, 2013; Stockburger, Schmälzle, Flaisch, Bublatzky, & Schupp, 2009) in visual cortical areas. Moreover, ERP amplitudes in response to food stimuli can be modulated via regulation, specifically in late time windows (Blechert, Feige, Hajcak, & Tuschen-Caffier, 2010; Sarlo et al., 2013; Svaldi et al., 2015). In particular, healthy female restraint eaters have shown reduced late ERP amplitudes towards high-calorie food pictures after regulation instructions (Svaldi et al., 2015). Restraint eaters have also shown reduced ERP amplitudes in response to available *v.* unavailable food, which might reflect uninstructed, implicit downregulation (Blechert et al., 2010). Taken together, ERP studies in healthy participants show attention biases towards food pictures in early and late time intervals, reflecting both early, more bottom-up driven automatic motivated attention and later, more top-down driven in-depth processing of motivationally relevant stimuli. In late time intervals, this can be influenced by regulation.

Despite the advantages of EEG/MEG, not many studies have used these techniques to investigate food processing in AN (Blechert, Feige, Joos, Zeeck, & Tuschen-Caffier, 2011; Godier, Scaife, Braeutigam, & Park, 2016; Nikendei et al., 2012; Novosel et al., 2014). Of the few studies that have used these techniques, the observed results point to different directions, as has been found in the behavioral and fMRI studies. Importantly, in early time intervals (*<*300 ms) neural responses towards food pictures are increased in AN patients compared to HC (Blechert et al., 2011; Godier et al., 2016). In late time intervals (*>*300 ms), such ERP amplitudes are either increased (Novosel et al., 2014), equally large (Godier et al., 2016) or reduced in AN patients (Nikendei et al., 2012). These conflicting results might partly be attributed to heterogeneous sample characteristics and research designs. However, the effects found in early time intervals suggest increased early motivated attention towards food in AN, and this might be regulated later on.

The aim of the current study is to use time-sensitive MEG to investigate motivational responses to food in AN. To this end, MEG was recorded in adolescent AN patients and age- and gender-matched HC during the passive viewing of high- and low-calorie food and non-food pictures. Similar to Godier et al. (2016), we used MEG-based source localization of neural activity in early and late time intervals. Following Blechert et al. (2011), we presented a multitude of stimuli in a passive viewing rapid serial visual presentation (RSVP) paradigm, but with a longer presentation time to ensure recognizability. Moreover, the non-food condition allowed us to disentangle differences in the motivational response to food in AN patients and HC from general illness-specific differences in neural functioning (Nikendei et al., 2012; Sfärlea et al., 2016).

This study explores whether AN patients show reduced (in line with most subjective reports) or increased (in line with their state of undernourishment) motivational responses to food. On the level of subjective experience, AN patients should rate food pictures as less palatable and report less craving towards food, especially for high-calorie food, as observed before (Lloyd & Steinglass, 2018). On the neurophysiological level, the motivational state should influence (reduce or increase) the effect of motivated attention in visual cortical areas, which is already at an early and automatic stage of processing (50–300 ms, Blechert et al., 2011). We speculate that the motivational response to food might be downregulated in AN via top-down processes in frontal regions (Hollmann et al., 2012) at later stages of processing (350–550 ms, Blechert et al., 2010). Moreover, successful downregulation might reduce the late effect of motivated attention in visual cortical regions in AN patients.

## Methods

### Participants

Patients with a main diagnosis of AN were recruited during inpatient treatment on the specialized ward for eating disorders at the Department of Child and Adolescent Psychiatry, University Hospital Muenster, Germany. Treatment comprised of a multimodal therapeutic concept according to the German S3-guidelines (Resmark, Herpertz, Herpertz-Dahlmann, & Zeeck, 2019) with a target weight at the 25th age-adjusted body mass index (BMI) percentile. Calories were gradually increased to about 2000–3000 kcal/day to achieve a weight gain of at least 500 g per week. All diagnoses were given by the treating clinician. In addition, AN diagnoses were confirmed by a clinical psychologist based on the Eating Disorder Examination Interview and Questionnaire (EDE-I and EDE-Q; Fairburn and Beglin, 1994) according to DSM-4 and reclassified according to DSM-5 (American Psychiatric Association, 2013). Patients with comorbid conditions were included, except for pervasive developmental disorders or psychotic disorders. Age- and gender-matched HC participants were recruited from secondary schools in Muenster. Participants reported no lifetime diagnosis of any mental disorder in a screening survey. HC participants with elevated EDE-Q scores and a BMI below the 25th or above the 90th age percentile were excluded. General exclusion criteria were intellectual disabilities, suicidality, substance abuse, somatic disorders with known influence on the central nervous system, pregnancy, and metallic implants or devices near the head (e.g. braces). All participants and their parents were informed of the study protocol in oral and written form and gave written informed consent. The local medical ethics committee approved this study.

### Stimuli

**Fig. 1.**
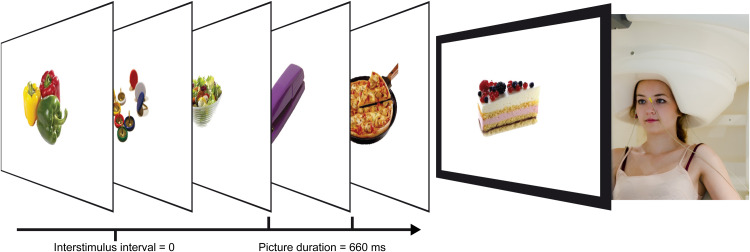
Participant in the MEG and RSVP paradigm. MEG, magnetoencephalography; RSVP, rapid serial visual presentation.

Three hundred pictures of food and non-food items were selected from the food-pics database, which contains pictures with normalized visual properties (brightness, complexity, and contrast) and nutrient information on the depicted food (Blechert, Meule, Busch, & Ohla, 2014; Fig. 1). As many of the AN patients (Heiss, Hormes, & Timko, 2017) and healthy adolescent girls (Patelakis et al., 2019) are vegetarian, we only included vegetarian food to avoid potentially confounding effects of participants’ dislike of meat. Food pictures were classified into 100 high-calorie and 100 low-calorie food pictures according to their calorie content per 100 g (High: *M* = 356.12, s.d. = 105.98; Low: *M* = 43.62, s.d. = 30.81; *t*(198) = 28.32, *p* *<* 0.001). The 100 non-food pictures included household objects, office supplies, tools and kitchen accessories.

### Procedure

MEG recordings took place approximately two hours after a midday meal, to control for satiation. AN patients ate according to their dietary plan on the ward and HC patients were asked to eat as usual. Participants were then introduced to the MEG chamber and the paradigm, where they reported the time of their last meal and answered the Food Cravings Questionnaire state version (FCQ-S, Cepeda-Benito, Gleaves, Williams, and Erath, 2000). After that, digital head renderings were recorded using a three-dimensional tracking device (Polhemus, 1993).

Participants were placed in the MEG scanner with their nasion 86 cm apart from a screen. Pictures were presented with a vertical visual angle of 9° while participants kept their eyes focused on a central red fixation cross. The RSVP paradigm presented pictures for 660 ms without inter-stimulus intervals in a pseudo-randomized manner (maximum three consecutive stimuli per category, equal transition probability) in two blocks, resulting in a total of 600 trials.

After the MEG recording, the 200 food pictures were presented again for 1500 ms in a pseudo-randomized order (as above) and rated with regard to palatability (How palatable do you find this food?) and craving (How much would you like to eat this food right now if it was in front of you?) on a contiguous analogue scale from 1 (not at all) to 9 (absolutely) (cf. Blechert et al., 2014). All stimulus presentations and picture ratings were performed using Presentation® Version 14.8 software (Neurobehavioral Systems, Inc., 2014).

### Behavioral data analysis

The ratings of palatability and craving were analyzed by repeated measures of analysis of variances (ANOVAs) with the factors food category (high calorie and low calorie) and group (AN, HC) and supplemented by *post hoc t* tests. All tests were performed using SPSS 22 (IBM Corp., 2013) and corrected for variance inhomogeneity if necessary.

### MEG data acquisition and analysis

Visually evoked magnetic fields (VEMF) were recorded using a 275-channel whole-head sensor system MEG with first-order axial SQUID gradiometers (Omega 275, CTF MEGTM, VSM Medtech Ltd.). Head position and movements were tracked using landmark coils in each ear channel and on the nasion. Continuous MEG data were recorded between 0 and 150 Hz using a sample rate of 600 Hz and then downsampled to 300 Hz. A zero-phase Butterworth second-order high-pass filter [12 dB/oct] and a fourth-order low-pass filter [24 dB/oct] with a cut-off frequency of 0.01 and 48 Hz were applied. Single-trial data editing and artifact rejection were conducted using the method for statistical control of artifacts in high-density MEG data (Junghoefer, Elbert, Tucker, & Rockstroh, 2000), identifying channel and global artifacts and substituting them via spline interpolation. Single epochs of 800 ms (200 ms before to 600 ms after stimulus onset) were averaged in correspondence to the experimental conditions. A pre-stimulus interval of 150 ms was used for baseline adjustment.

Estimation of neural sources underlying the VEMFs was done using the L2-Minimum-Norm approach (L2-MNE; Hämäläinen and Ilmoniemi, 1994) with a spherical shell consisting of 350 evenly distributed dipole pairs as the source model. A source shell radius of 87% of the individually fitted head radius was chosen, which roughly corresponds to the grey matter depth. Leadfield matrices were calculated for all participants and conditions using a Tikhonov regularization parameter lambda of 0.1. The estimated neural activity was calculated as vector length of each dipole pair. Topographic maps of the estimated neural activity—displaying the direction-independent current dipole activity (Junghoefer et al., 2010)—were calculated for each participant, condition, and time point based on averaged magnetic field distributions and individual sensor positions. For visualization purposes, L2-MNE results were projected onto a model brain.

Statistical MEG data analyses calculated repeated ANOVA measures with the factors food category (high calorie, low calorie, non-food) and Group (AN, HC) for each dipole and time point. Statistical analyses comprised two separate time intervals of interest (TOIs) with 50 ms gaps to exclude effects of picture onset and offset and to clearly differentiate early (50–300 ms) from late effects (350–550 ms). Corrections for multiple comparisons used nonparametric testing procedures, similar to the cluster-mass test used for analyzing fMRI data (Maris & Oostenveld, 2007). Within each TOI, estimated sources were considered for further analysis only if they showed significant ANOVA effects surpassing *p* values of *<*0.01 (first-level criterion). Temporally and/or spatially adjacent first-level significant *F* values of the underlying sources forming spatio-temporal clusters were then summed to constitute the cluster masses. Cluster masses were evaluated against distribution of 1000 random permutations of the same data sets (for each permutation, the biggest identified first-level significant cluster mass was considered). Clusters were only considered significant if their cluster mass surpassed the 950th highest cluster mass of the random distribution, equivalent to *p* *<* 0.05 (second-level criterion). Preprocessing and analysis of MEG data used the MATLAB-based software EMEGS Version 3.1 (The MathWorks Inc, 2014; emegs.org; Peyk, De Cesarei, and Junghoefer, 2011).

## Results

### Subjects’ characteristics

A total of 22 AN patients and 29 HC participants were recruited. One AN patient was excluded due to abnormal clinical magnetic resonance imaging findings, and one HC participant due to a high BMI. The final analysis included 21 adolescent patients with restrictive type AN and 28 HCs. All participants attended secondary schools and the majority of both groups prepared for a general qualification for university entrance (AN: 71.4%, HC: 89.3%). AN and HC did not differ in age, time since last meal and state of food craving (FCQ-S, Table 1). Compared to HC, AN patients had significantly lower BMIs and higher self-reported eating disorder symptoms (EDE-Q). AN patients were recruited during ongoing treatment (days since admission *M* = 53.9, s.d. = 29.26, range: 14–116 days) and after partial weight restitution (BMI change since admission *M* = 0.89, s.d. = 1.20, range: −0.63–4.47)^†^1^^. Still, 19 AN patients had BMIs below the 10th age percentile. HC participants had BMIs between the 26th and 67th age percentile. In the AN group, comorbid conditions were depression (*N* = 5), social anxiety disorder (*N* = 2), and generalized anxiety disorder (*N* = 1). Four patients were taking psychotropic medications (2 Olanzapin, 1 Escitalopram, and 1 Mirtazapin). The average illness duration was ~17 months (*M* = 532.14 days, s.d. = 414.08). Twelve AN patients were in their first inpatient treatment, whereas nine AN patients had one or more prior inpatient treatments (for exploratory correlation analyses between subject characteristics and experimental data, see online Supplementary Table S1).

**Table 1.** Subjects’ Characteristics. Data Captured at the Time of MEG recording unless noted

	AN [*N* = 21]	HC [*N* = 28]	*t*(*df*)	*p*	
AGE	15.51 ± 1.89	15.07 ± 1.96	*t*(47) = 0.77	*p* = .441	n.s.
TIME since last meal	2:24 ± 0:17	2:37 ± 0:47	*t*(35.67) = −1.35	*p* = .184	n.s.
FCQ-S	1.97 ± 0.82	2.13 ± 0.67	*t*(46) = −0.71	*p* = .479	n.s.
BMI	15.91 ± 1.57	19.72 ± 2.28	*t*(47) = −6.89	*p* *<* 0.001	AN *<* HC
BMI-Percentile	6.97 ± 10.07	46.39 ± 20.68	*t*(41.2) = −8.79	*p* *<* 0.001	AN *<* HC
BMI-SDS	−2.18 ± 1.21	−0.06 ± 0.67	*t*(29.21) = −7.21	*p* *<* 0.001	AN *<* HC
EDE-Q^1^ total	3.22 ± 1.58	0.81 ± 0.81	*t*(24.03) = 6.05	*p* *<* 0.001	AN *>* HC
EDE-Q restrained	3.14 ± 2.14	0.43 ± 0.58	*t*(19.88) = 5.95	*p* *<* 0.001	AN *>* HC
EDE-Q eating	2.40 ± 1.32	0.35 ± 0.69	*t*(24.64) = 6.44	*p* *<* 0.001	AN *>* HC
EDE-Q weight	3.31 ± 1.81	1.19 ± 1.16	*t*(27.13) = 4.21	*p* *<* 0.001	AN *>* HC
EDE-Q shape	4.01 ± 1.63	1.31 ± 1.24	*t*(30.62) = 5.81	*p* *<* 0.001	AN *>* HC
DAYS since admission	53.9 ± 29.26				
BMI change since admission	0.89 ± 1.20				

BMI, body mass index; BMI-Percentile, body mass index age percentile; BMI-SDS, body mass index standard deviation scores; FCQ-S, Food Cravings Questionnaire—State (absolute values from 0 to 5); EDE-Q, Eating Disorder Examination Questionnaire; MEG, magnetoencephalography.

### Ratings

**Fig. 2.**
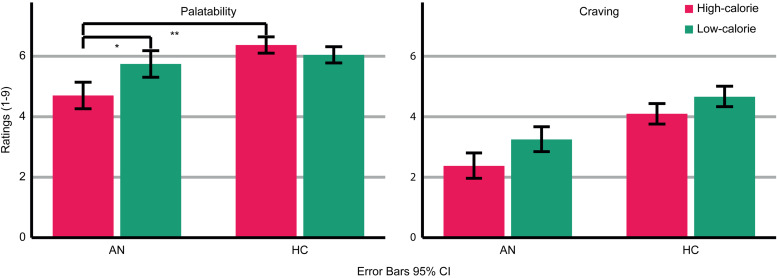
Ratings of palatability and craving for the depicted high- and low-calorie food using a scale from 1 (not at all) to 9 (absolutely). Main effects of group and food category were found for palatability and craving ratings. An interaction occurred for palatability ratings. Asterisks indicate the most relevant significant effects.

AN patients rated the depicted food as less palatable than HC (Group: *F*_(1,47)_ = 9.793; *p* *=* 0.003; *η*_p_^2^ = 0.172 Fig. 2). Across groups, palatability ratings were comparable for high- and low-calorie food (food category: *F*_(1,47)_ = 2.294; *p* *=* 0.137; *η_p_*^2^ = 0.047). However, a significant interaction (Food-Category × Group: *F*_(1,47)_ = 8.374; *p* *=* 0.006; *η*_p_^2^ = 0.151) indicated that AN patients rated high-calorie food as less palatable than low-calorie food (AN High *v.* Low *t*(20) = 2.479; *p* *=* 0.022), whereas HC rated high- and low-calorie food as similarly palatable (HC High *v.* Low: *t*(27) = 1.245; *p* *=* 0.224). Palatability ratings did not differ between groups regarding low-calorie food (*t*(47) = −0.858; *p* *=* 0.395) but did regarding high-calorie food (*t*(22.977) = −3.416; *p* *=* 0.002). Thus, palatability ratings were specifically reduced for high-calorie food in AN patients.

AN patients reported less craving for the depicted food than HC (Group: *F*_(1,47)_ = 12.644; *p* *=* 0.001; *η_p_*^2^ = 0.212). Across groups, more craving was reported for low-calorie compared to high-calorie food (Food-Category: *F*_(1,47)_ = 7.981; *p* *=* 0.007; *η*_p_^2^ = 0.145). There was no significant interaction (Food-Category × Group: *F*_(1,47)_ = 0.344; *p* *=* 0.560; *η*_p_^2^ = 0.007).

### MEG results

**Fig. 3.**
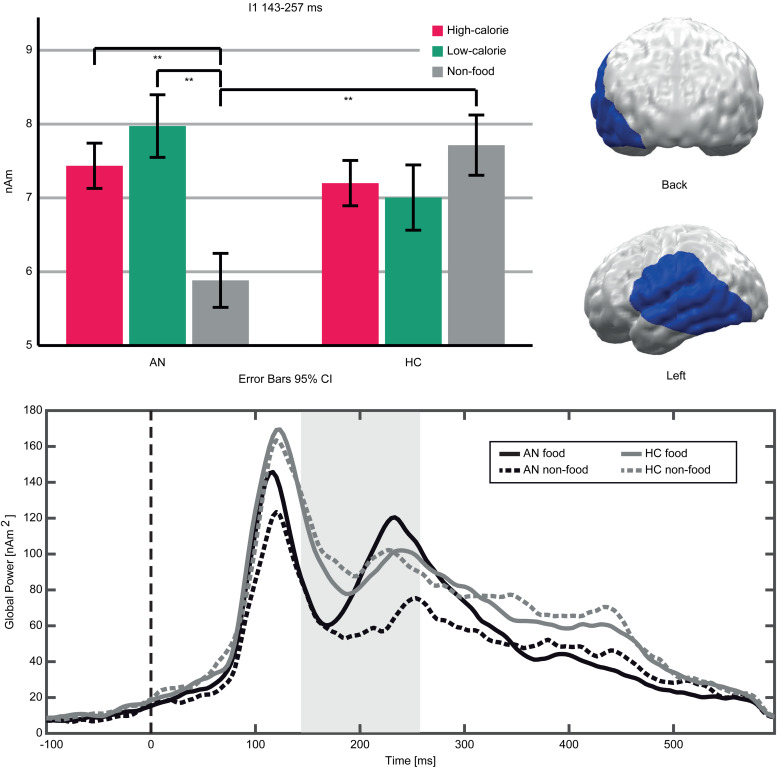
Top left: Bar graphs of estimated neural activity [nAm] for the Food-Category × Group interaction in Cluster I1. Top right: Topographic representation of Cluster I1. Bottom: Global power plot of estimated neural activity [nAm2] in Cluster I1 in response to food (high- and low-calorie food averaged for visualization purposes) and non-food pictures in AN and HC. Significant time interval marked in grey.

An interaction (Food-Category × Group) in the early TOI indicated stronger neural responses to food compared to non-food pictures in AN patients *v.* HC (Fig. 3). The respective cluster I1 (143–257 ms, *p* cluster*<*0.001, *F*_(2,94)_ = 19.623, *p* < *0*.001; *η*_p_^2^ = 0.295) was located in the left occipito-temporal cortex and inferior frontal gyrus (IFG). AN patients showed stronger neural activity in response to both high- and low-calorie food compared to non-food pictures (AN High *v.* Non-food: *t*(20) = 6.16, *p* < *0*.001; AN Low *v.* Non-Food: *t*(20) = 5.97, *p* < *0*.001). In this cluster, HC showed no significant differences between food and non-food pictures (HC High *v.* Non-food: *t*(27) = −1.84, *p* *=* 0.076; HC Low *v.* Non-Food: *t*(27) = −1.83, *p* *=* 0.077). In both groups, neural activity in response to high- and low-calorie food pictures did not differ (AN High *v.* Low: *t*(20) = −1.74, *p* *=* 0.096; HC High *v.* Low: *t*(27) = 0.62, *p* *=* 0.537). Separate comparisons of picture categories between groups revealed that non-food pictures elicited lower neural activity in AN *v.* HC, whereas neural responses to both food categories did not differ (High AN *v.* HC: *t*(47) = 0.29, *p* *=* 0.771; Low AN *v.* HC: *t*(47) = 1.15, *p* *=* 0.255; Non-food AN *v.* HC: *t*(47) = −2.12, *p* *=* 0.039). In the late TOI, no significant interaction emerged.

**Fig. 4.**
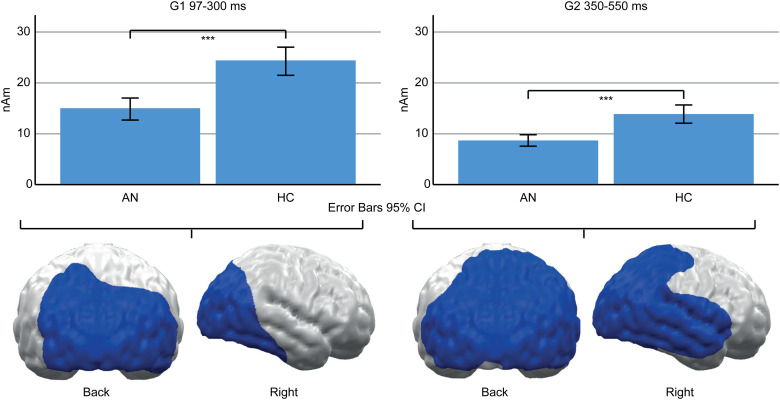
Top: Bar graphs of estimated neural activity [nAm] for the main effect group in early Cluster G1 (left) and late G2 (right). Bottom: Topographic representation of cluster G1 (left) and G2 (right).

A main effect of Group (AN, HC) indicated lower neural activity in AN patients compared to HC in both TOIs (Fig. 4). In the early TOI, this was observed in cluster G1 (97–300 ms; *p* cluster*<*.001, *F*_(1,47)_ = 27.332, *p* < .001; *η*_p_^2^ = 0.368), located in bilateral occipital and right temporal cortices. In the late TOI, the same effect was found in cluster G2 (350–550 ms; *p* cluster = .002; *F*_(1,47)_ = 21.755, *p* <.001; *η*_p_^2^ = 0.316), located in similar regions but with further extension to the right temporal and parietal cortices.

The main effect of food category (high calorie, low calorie, and non-food) indicated stronger neural responses to food pictures specifically in the early TOI in both groups. These findings are reported in detail in the supplement (online Supplementary Fig. S1) but are summarized here: in the early TOI, four clusters (F1–F4) revealed increased neural activity in response to food compared to non-food pictures, starting in the bilateral OCC and spreading to temporal, parietal, and frontal areas. Two of the clusters (F2, F3) additionally revealed stronger neural activity in response to low- compared to high-calorie food. In the late TOI, neural activity was conversely lower specifically in response to a low-calorie food. Two clusters (F5, F6) revealed reduced neural activity in temporal, parietal, and frontal areas in response to low-calorie food pictures compared to both other categories. Neural responses to high-calorie food pictures were either similar or stronger compared to non-food pictures.

## Discussion

The present study used time-sensitive MEG to investigate the motivational response to food in adolescent AN patients compared to HC. On the level of subjective experience, AN patients showed the expected reduced ratings of their appetitive motivational response to the depicted food. In contrast, on the neurophysiological level, AN patients showed a relatively increased neural response to food *v.* non-food pictures during an early, more automatic stage of processing. This effect was no longer observed in a late stage of processing.

With respect to their subjective experience, AN patients compared to HC rated high-calorie food as less palatable and reported feeling less craving towards all kinds of food. This is consistent with prior studies (Lloyd & Steinglass, 2018) and would be in line with an overall reduced motivational response to food in AN. Both AN patients and HC reported more craving for low-calorie food. This is consistent with normative ratings (online Supplementary material) and presumably reflects that the selected low-calorie foods were rather attractive. However, only AN patients rated palatability higher for low-calorie than high-calorie food. Relatively more positive ratings of low- *v.* high-calorie food have been suggested to reflect dietary rules related to the fear of gaining weight (Cowdrey et al., 2013; Jiang et al., 2010).

In contrast to their self-reports, AN patients’ early neural responses to food pictures were relatively increased. Although clusters in adjacent regions and time windows showed reduced neural activity in AN patients (G1, G2, Fig. 4) and a preference for food *v.* non-food pictures in both groups (F1–F4, online Supplementary Fig. S1), cluster I1 (Fig. 3) showed a relatively increased neural response to food stimuli only in AN. This converges with prior findings (Blechert et al., 2011; Godier et al., 2016) and presumably reflects bottom-up driven, automatic motivated attention (Pourtois et al., 2013; Stockburger et al., 2008). Consistent with the common view of food as an appetitive stimulus, it is suggested that not only acute (Stockburger et al., 2008) but also chronic starvation enhances the effect of motivated attention towards food. Yet, the motivational value of food might be less clear in AN: It could be affected by conflicting aversive feelings or a general preoccupation with food (Fürtjes et al., 2018, 2020). Still, strong main effects of food *v.* non-food (online Supplementary Fig. S1) argue against a qualitative difference between HC and AN. Perhaps more importantly, in a state of undernourishment, early, automatic attention towards food might be an adaptive and possibly survival-promoting mechanism.

Interestingly, the increased early neural response to food *v.* non-food pictures included not only visual areas but also left frontal areas (F3, 207–277 ms; F4, 233–300 ms), starting even earlier in AN patients (IFG part of cluster I1, 143–257 ms). The opercular IFG, as part of the primary gustatory cortex, responds to visual food stimuli (Yousuf, Heldmann, Göttlich, Münte, & Doñamayor, 2018). Thus, our results might indicate that enhanced early motivated attention towards food pictures activates not only visual but also gustatory regions. On the other hand, frontal activity is typically not associated with bottom-up but with top-down processes (Buschman & Miller, 2007), and these were expected later. In the late time interval, however, food-related group differences disappeared. As the IFG has also been related to downregulation of appetitive food responses (Hollmann et al., 2012) and resistance to food desire in everyday eating behavior (Lopez, Hofmann, Wagner, Kelley, & Heatherton, 2014), the observed early frontal activity might also be associated with rapid downregulation.

Beyond the observed food-related effects, neural activity in the posterior cortex was reduced in AN patients (G1, G2, Fig. 4). This converges with reduced ERP amplitudes in AN patients (Hatch et al., 2010; Li et al., 2015a, 2015b; Nikendei et al., 2012; Pollatos, Herbert, Schandry, & Gramann, 2008; Sfärlea et al., 2016) and may be related to brain atrophy (Seitz et al., 2013). Such reduced amplitudes might reflect either reduced neural functioning or measurement artifacts, e.g. due to the increased CSF-Volume (EEG) or distance between brain and sensors (MEG; Vorwerk et al., 2014). However, AN patients were partially weight restored, and brain structure might normalize rapidly with weight gain (Bernardoni et al., 2016). Unfortunately, brain structure was not measured in this study and, thus, this interpretation remains speculative.

The present study is limited by several methodological constraints. First, the study included adolescent restrictive-type AN patients during ongoing treatment, in some cases after partial weight restoration. Thus, conclusions cannot necessarily be generalized to adult patients, patients with binge–purge type AN, or severely underweight patients under current food restriction. However, the focus on a relatively homogenous sample with a small age range and little comorbidity might, despite its small size, strengthen the informative value for this specific group. Future research should investigate AN patients at different states of starvation. Second, the passive viewing RSVP paradigm allows only speculative interpretation of the processes underlying the observed neurophysiological effects (e.g. automatic downregulation), as this cannot be inferred from the performed task. Moreover, this paradigm has comparatively low ecological validity. Nonetheless, the paradigm was chosen because it presents many stimuli in a short time without any task-specific cognitive load. This allows one to investigate the spontaneous response to food stimuli with a good signal-to-noise ratio. Third, because many AN patients (Heiss et al., 2017) and healthy adolescent girls (Patelakis et al., 2019) are vegetarian, only vegetarian food was presented. This limits comparability with previous studies, which have typically included meat. Finally, AN patients might also show aversive or anxious responses to food (Neimeijer et al., 2017). As the effect of motivated attention is similar for both appetitive and aversive stimuli (Lang & Bradley, 2010), the observed neurophysiological effects might also reflect (at least in part) aversive responses to food in AN patients. However, food aversion is unlikely in HC participants. Thus, the fact that both groups show largely similar neurophysiological effects, including relatively higher ratings and correspondingly stronger neurophysiological responses to low- *v.* high-calorie food (F2, F3, online Supplementary Fig. S1), suggests essentially appetitive food responses. Still, future studies should ask participants to rate the presented pictures not only regarding their appetitive but also their aversive value.

In sum, this study revealed that adolescent restrictive type AN patients showed a relatively increased early neural response to food pictures, despite their reduced self-reported appetitive value. This speaks against an overall reduced motivational response to food in AN. Conversely, the early onset of an increased neurophysiological response suggests enhanced automatic motivated attention to food, arguably an adaptive mechanism in a state of undernourishment. Such AN-related early motivated attention effects were no longer present shortly afterwards, implying spontaneous, implicit downregulation. This might be interpreted as the result of a strong habitual suppression of the desire to eat in AN. Thus, in AN, the conflicting tendencies of automatic physiological approach and rapid downregulation might not be under deliberate control and, thus, may be difficult to influence via therapeutic strategies.

## Footnotes

† The notes appear after the main text.

## Footnotes

1 EDE-I and EDE-Q were conducted within ten days of the MEG measurement (*M* = 9.57, s.d. = 10.25 days). To clarify the fulfillment of diagnostic criteria at the beginning of treatment and independent of the current dietary plans, patients were asked about their eating disorder symptoms in the 28 days prior to their admission. DSM-4 and DSM-5 criteria for restrictive AN were fulfilled in all cases. It can be assumed that many patients achieved partial remission.

## Supplementary material

For supplementary material accompanying this paper visit https://doi.org/10.1017/S003329172100088X. click here to view supplementary material
