# Supplementary material for: Increased early motivational response to food in adolescent anorexia nervosa revealed by magnetoencephalography
Source: Psychol Med. 2021 May 5;52(16):4009–17. doi: 10.1017/S003329172100088X (PMC9811273; doi:10.1017/S003329172100088X)
Supplement: Supplementary file 1 [file S003329172100088Xsup001.pdf]

Supplementary material

Main effect of Food-Category (High-calorie, Low-calorie, Non-food)

In the early TOI, analyses revealed four clusters (F1-F4; Figure S1) with a main effect of Food-Category (High-calorie, Low-calorie, Non-food). In cluster F1 (77-150ms; cluster significance *p*-cluster<.001; $F(2,94)=78.089$, $p<.001$; $\eta_p^2=0.624$), located in the OCC, both high- and low-calorie food pictures elicited higher neural activity than non-food pictures (High vs. Non-food: $t(48)=9.91$, $p<.001$; Low vs. Non-food: $t(48)=8.75$, $p<.001$). Neural responses to high- compared to low-calorie food pictures did not differ ($t(48)=.691$, $p=.493$). In cluster F2 (170–300ms; *p*-cluster<.001; $F(2,94)=26.962$, $p<.001$; $\eta_p^2=0.365$), located in the occipital, right temporal and medial parietal cortex, both kinds of food pictures elicited higher neural activity compared to non-food pictures (High vs. Non-food: $t(48)=4.29$, $p<.001$; Low vs. Non-food: $t(48)=6.22$, $p<.001$). Moreover, high-calorie food pictures elicited lower neural activity than low-calorie food pictures ($t(48)=-4.71$, $p<.001$). In cluster F3 (207–277ms; *p*-cluster<.01; $F(2,94)=26.631$, $p<.001$; $\eta_p^2=0.362$), located in the left temporal and frontal cortex, effects resembled cluster F2: Both kinds of food pictures elicited higher neural activity compared to non-food pictures (High vs. Non-food: $t(48)=2.80$, $p=.007$; Low vs. Non-food: $t(48)=6.03$, $p<.001$) and high-calorie food pictures elicited lower neural activity than low-calorie food pictures (High vs. Low: $t(48)=-4.17$, $p<.001$). In cluster F4 (233-300ms; *p*-cluster=.034; $F(2,94)=11.969$, $p<.001$; $\eta_p^2=0.203$), located in the left orbitofrontal cortex and temporal pole, effects resembled cluster F1: both high- and low-calorie food pictures elicited higher neural activity than non-food pictures (High vs. Non-food: $t(48)=2.89$, $p=.006$; Low vs. Non-food: $t(48)=5.37$, $p<.001$). Neural responses to high- vs. low-calorie food pictures did not differ ($t(48)=-1.23$, $p=.221$).

In the late TOI, analyses revealed two clusters with a significant main effect of Food-Category (F5, F6). In cluster F5 (300–550ms; *p*-cluster<.001; $F(2,94)=34.245$, $p<.001$; $\eta_p^2=0.422$), located in prefrontal, midline fronto-parietal and right temporal cortical regions, neural activity was specifically reduced in response to low-calorie food pictures (High vs. Low: $t(48)=7.80$, $p<.001$; Low vs. Non-food: $t(48)=-7.02$, $p<.001$). Neural responses to high-calorie food and non-food pictures did not differ ($t(48)=-1.18$, $p=.242$). In cluster F6 (413-550ms; *p*-cluster<.005; $F(2,94)=12.947$, $p<.001$; $\eta_p^2=0.216$), located in the left occipito-temporal cortex, high-calorie food pictures elicited stronger neural activity than both low-calorie food ($t(48)=4.40$, $p<.001$) and non-food pictures ($t(48)=3.11$, $p=.003$). Moreover, similar to cluster F5, neural activity in response to low-calorie food pictures was lowest (Low vs. Non-food: $t(48)=-2.46$, $p=.018$).

Figure S1.

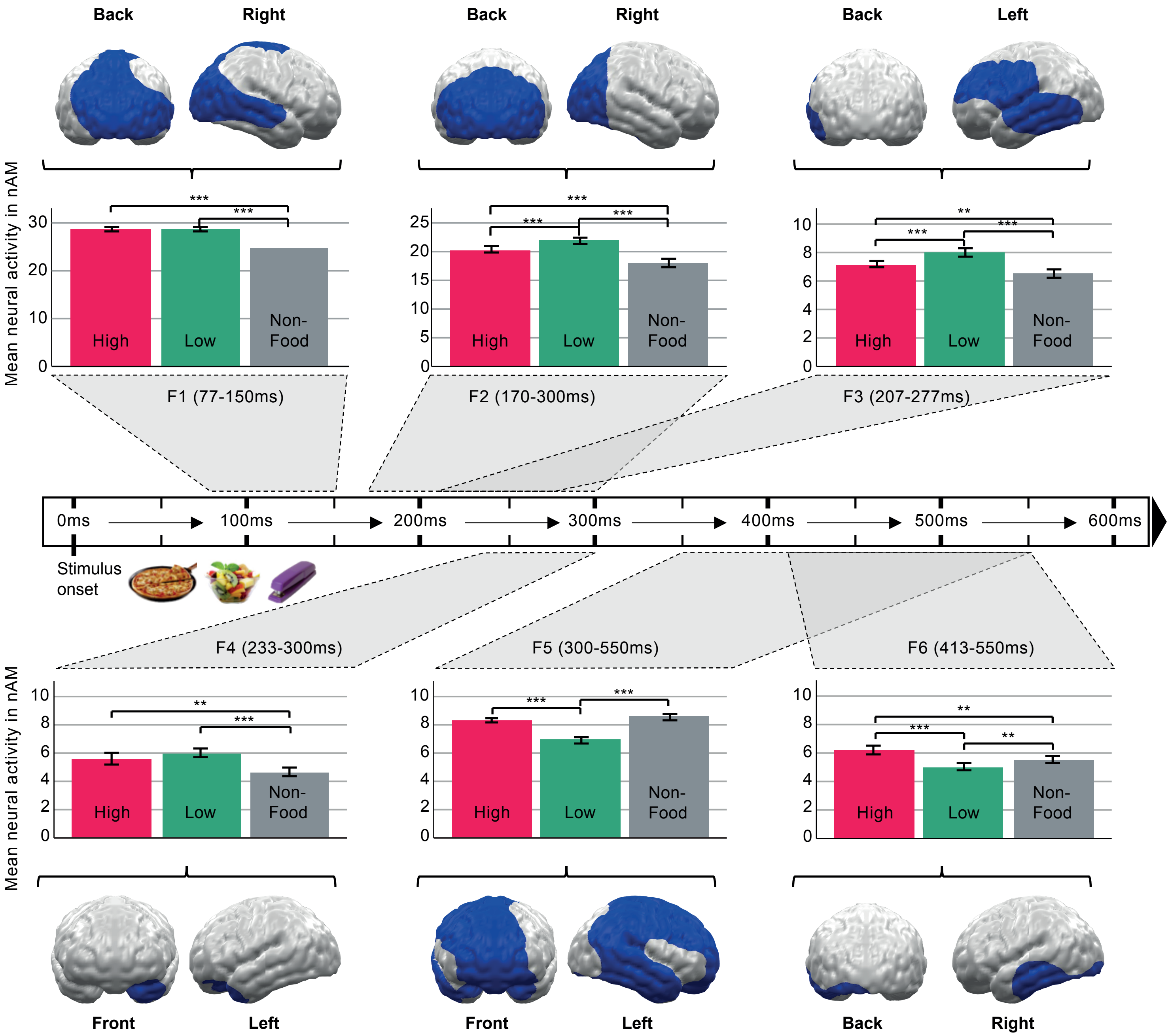

Error bars 95% CI

Figure S1. Bar graphs of estimated neural activity [nAm] for the main effect of Food-Category and topographic representations of Clusters F1-F6.

Additional cluster analysis

As an additional cluster analysis strategy, we first looked for clusters with a significant main effect of Food-Category in HC only (i.e. the main regions of food processing unaffected by AN). In the next step, the obtained clusters were applied to the AN group data and the respective estimated neural activity was exported from the MATLAB-based software EMEGS Version 3.1 (emegs.org; Peyk et al., 2011) to SPSS 22 (IBM, Armonk, N.Y.). Finally, we calculated an ANOVA with the factors Food-Category (High-calorie, Low-calorie, Non-food) and Group (AN, HC).

The first step of this analysis (HC only) revealed two clusters in the early (HCF1, HCF2) and three clusters in the late TOI (HCF3, HCF4, HCF5). The ANOVA including both groups (AN, HC) revealed significant main effects of Food-Category in all 5 clusters that overall replicated the findings of clusters F1, F2 and F5 of the original analysis (Figure S2, cf. Figure S1). Clusters F3, F4 and F6 were not present in this additional analysis, presumably because the use of a smaller sample for the cluster selection (HC only) reduced statistical power. Furthermore, the ANOVAs revealed no interaction of Food-Category x Group in any of the clusters (Table S1). There was one trend-level interaction effect in cluster HCF3, with a less pronounced reduction of activity in response to Low-calorie food pictures in AN patients.

The main finding of the original cluster analysis was enhanced neural processing in response to food vs. non-food pictures in cluster I1 (Figure 3, main text) specifically in AN, but not in HC. Accordingly, this additional cluster analysis based on the main effect of Food-Category in HC only did not reveal a cluster corresponding to cluster I1. We suggest that processes of motivated attention affected neural activity in partly different regions and time points in AN patients and HCs. Therefore, this analysis tailored to clusters in which HCs showed the most significant effects overlooked presumably functionally similar effects in slightly different locations/time windows in AN patients.

Figure S2.

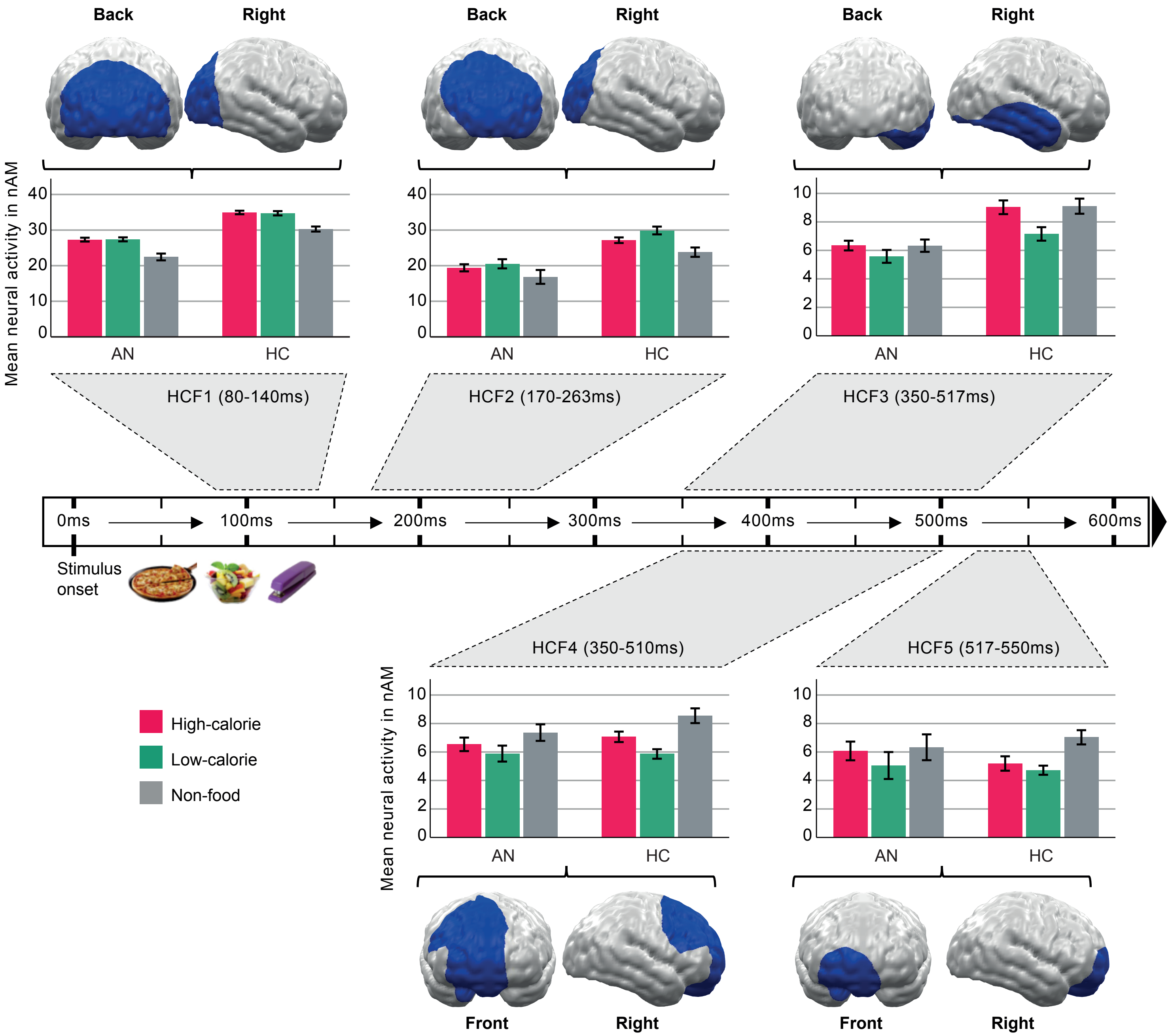

Error bars 95% CI

Figure S2. Bar graphs of estimated neural activity [nAm] for the main effect of Food-Category divided in groups (AN, HC) and topographic representations of Clusters HCF1-HCF5 based on the main effect of Food-Category for HCs.

Table S1

Additional cluster analysis

TOI	Cluster	*Effect*	*(df)*	*F*	*p*	η^2
50-300ms	HCF1 (80-140ms)	Food-Category	2, 94	96.782	<.001	.673
		Food-Category x Group	2, 94	0.222	.801	.005
	HCF2 (170-263ms)	Food-Category	2, 94	21.690	<.001	.316
		Food-Category x Group	2, 94	1.346	.265	.028
350-550ms	HCF3 (350-517ms)	Food-Category	2, 94	16.256	<.001	.257
		Food-Category x Group	2, 94	3.080	.051	.062
	HCF4 (350-510ms)	Food-Category	2, 94	27.851	<.001	.372
		Food-Category x Group	2, 94	2.360	.100	.048
	HCF5 (517-550ms)	Food-Category	2, 94	11.608	<.001	.198
		Food-Category x Group	2, 94	2.294	.106	.047

ANOVA results of the additional cluster analysis. Lines in white show the main effect Food-Category (High-calorie, Low-calorie, Non-food), Lines in Grey the interaction Food-Category x Group (AN, HC).

Normative Picture Ratings

According to the normative ratings given in the food-pics database (rating scale: 0-100, Blechert et al., 2014), the selected 100 high-calorie, 100 low-calorie, and 100 non-food pictures obtained comparable recognizability ratings that were all above 97% (High-calorie: M=97.54, SD=4.68, range: 78.6 to 100; Low-calorie: M=98.78, SD=3.31, range: 75.4 to 100; Non-food: M=98.34, SD=3.68, range: 79.5 to 100; $F(2,297)=2.573$, $p<.05$). Moreover, the selected food pictures obtained higher normative ratings for pictures of low-calorie compared to high-calorie foods regarding both palatability (Low-calorie: M=66.81, SD=10.50; High-calorie: M=60.96, SD=7.87; $t(183.53)=4.460$; $p<.001$) and craving (Low-calorie: M=41.69, SD=13.44; High-calorie: M=35.33, SD=7.68; $t(157.43) = 4.110$; $p<.001$).

Explorative Correlation Analyses

The estimated cortical activity for the spatio-temporal cluster I1 with significant interaction effects of Group and Food-Category was exported from the MATLAB-based software EMEGS Version 3.1 (emegs.org; Peyk et al., 2011) to SPSS 22 (IBM, Armonk, N.Y.). The frontal part of the cluster I1, most likely to represent the IFG, was manually selected and its estimated cortical activity exported. Non-food mean activity was subtracted from each of both food categories. Explorative linear correlation analyses were performed within the AN patients' group only. We explored correlations of palatability and craving ratings with eating disorder symptoms (EDE-Q and subcategories restrained eating (r), eating concern (e), weight concern (w) and shape concern (s)) and illness duration. Also, the mean cortical activity was correlated with eating disorder symptoms (EDE-Q) as well as with illness duration. No significant correlation was observed between ratings, neural activity and EDE-Q values or illness duration (Table S2).

Table S2

Explorative Correlation Analyses

**Ratings**		**EDE-Q total**	**EDE-Q restricted**	**EDE-Q eating**	**EDE-Q weight**	**EDE-Q shape**	**Illness Duration**
Palatability	*r*	.19	.01	.30	.24	.20	-.25
	p	.403	.934	.187	.287	.385	.266
Craving	*r*	-.20	-.32	-.14	-.09	-.19	-.14
	p	.372	.154	.543	.685	.405	.541
**Neural activity**							
**Cluster I1**		**EDE-Q total**	**EDE-Q restricted**	**EDE-Q eating**	**EDE-Q weight**	**EDE-Q shape**	**Illness Duration**
High-calorie	*r*	.14	.21	.06	.22	-.01	-.10
	p	.533	.360	.774	.332	.963	.656
Low-calorie	*r*	.20	.25	.15	.23	.07	.08
	p	.379	.265	.500	.313	.748	.71
Non-food	*r*	.20	.22	.22	.24	.05	-.18
	p	.367	.321	.331	.282	.809	.417
High minus Non-food	*r*	-.08	.00	-.26	-.00	-.12	.13
	p	.706	.977	.241	.992	.600	.571
Low minus Non-food	*r*	.07	.14	.02	.07	.05	.38
	p	.737	.542	.926	.737	.814	.087
**IFG**							
High-calorie	*r*	.27	.24	.32	.28	.17	-.14
	p	.227	.291	.155	.212	.445	.541
Low-calorie	*r*	.36	.37	.40	.28	.28	.11
	p	.107	.098	.068	.212	.214	.623
Non-food	*r*	.30	.26	.39	.27	.23	-.14
	p	.173	.251	.076	.234	.303	.531
High minus Non-food	*r*	-.04	-.02	-.12	.05	-.11	-.00
	p	.843	.931	.578	.805	.625	.978
Low minus Non-food	*r*	.22	.30	.18	.13	.18	.40
	p	.334	.181	.432	.559	.431	.069

Explorative correlation analysis in AN patients (n=21). Pearson correlation (r) in white rows, 2-tailed Significance in grey rows.
